# Macrophage‐derived extracellular vesicles alter cardiac recovery and metabolism in a rat heart model of donation after circulatory death

**DOI:** 10.1111/jcmm.18281

**Published:** 2024-04-23

**Authors:** Selianne Graf, Vanessa Biemmi, Maria Arnold, Adrian Segiser, Anja Müller, Natalia Méndez‐Carmona, Manuel Egle, Matthias Siepe, Lucio Barile, Sarah Longnus

**Affiliations:** ^1^ Department of Cardiac Surgery Inselspital Bern University Hospital, University of Bern Bern Switzerland; ^2^ Department for BioMedical Research University of Bern Bern Switzerland; ^3^ Graduate School of Cellular and Biomedical Sciences University of Bern Bern Switzerland; ^4^ Laboratory for Cardiovascular Theranostics Cardiocentro Ticino Institute‐EOC Lugano Switzerland

**Keywords:** cardiac ischaemia–reperfusion injury, cardiac glucose metabolism, DCD heart transplantation, ex situ heart perfusion/ex vivo heart perfusion, macrophage‐derived extracellular vesicles

## Abstract

Conditions to which the cardiac graft is exposed during transplantation with donation after circulatory death (DCD) can trigger the recruitment of macrophages that are either unpolarized (M0) or pro‐inflammatory (M1) as well as the release of extracellular vesicles (EV). We aimed to characterize the effects of M0 and M1 macrophage‐derived EV administration on post‐ischaemic functional recovery and glucose metabolism using an isolated rat heart model of DCD. Isolated rat hearts were subjected to 20 min aerobic perfusion, followed by 27 min global, warm ischaemia or continued aerobic perfusion and 60 min reperfusion with or without intravascular administration of EV. Four experimental groups were compared: (1) no ischaemia, no EV; (2) ischaemia, no EV; (3) ischaemia with M0‐macrophage‐dervied EV; (4) ischaemia with M1‐macrophage‐derived EV. Post‐ischaemic ventricular and metabolic recovery were evaluated. During reperfusion, ventricular function was decreased in untreated ischaemic and M1‐EV hearts, but not in M0‐EV hearts, compared to non‐ischaemic hearts (*p* < 0.05). In parallel with the reduced functional recovery in M1‐EV versus M0‐EV ischaemic hearts, rates of glycolysis from exogenous glucose and oxidative metabolism tended to be lower, while rates of glycogenolysis and lactate release tended to be higher. EV from M0‐ and M1‐macrophages differentially affect post‐ischaemic cardiac recovery, potentially by altering glucose metabolism in a rat model of DCD. Targeted EV therapy may be a useful approach for modulating cardiac energy metabolism and optimizing graft quality in the setting of DCD.

## 1 INTRODUCTION

Although hearts obtained with donation after circulatory death (DCD) are subjected to potentially damaging periods of haemodynamic instability followed by warm ischaemia and reperfusion prior to procurement, ex‐situ heart perfusion (ESHP) has been used to limit graft damage and reported recipient outcomes are excellent. According to first results, DCD hearts provide similar 5‐year outcomes in terms of graft survival, rejection episodes and hospital stay when compared to cohorts of heart transplant patients receiving grafts obtained with conventional donation after brain death.^1^, ^2^ Furthermore, the use of DCD hearts has increased transplantation activity by 26%–48%.^3^, ^4^

Now that the feasibility and benefits of DCD heart transplantation have been demonstrated,^5^ research is required to optimize clinical protocols. For example, cardioprotective strategies upon reperfusion could be applied to limit graft injury and improve graft quality. Targeting circulating extracellular vesicles (EV) has been proposed as one such therapeutic strategy during ESHP.^6^ EV are released from many cell types^7^ and stress, such as ischaemia and reperfusion,^8^ alters EV release, as well as their cargo. In the context of DCD heart transplantation, conditions related to donor death affect molecular and immunologic properties of the secreted EV.^9^ Interestingly, administration of EV from cardiac progenitor cells, bone marrow stem cells as well as plasma from healthy rats and humans have been demonstrated as cardioprotective in the setting of ischaemia–reperfusion injury (IRI).^10^, ^11^ However, EV released during IRI have also been reported to mediate an inflammatory response by mobilizing monocytes^12^ and to impede organ recovery.^13^

Although little is known about macrophage infiltration in DCD, monocytes as well as unpolarized and pro‐inflammatory macrophages are expected to be present during ESHP; originating from both donor blood (used also in the perfusate during ESHP) and tissue‐resident cells. Indeed, brain death and prolonged cold ischaemia trigger sterile inflammation in heart transplantation^14^ and inflammatory and stress responses are induced during ESHP.^15^ Similar to myocardial infarction, pro‐inflammatory macrophages (M1) are expected to infiltrate the heart and tissue‐resident, unpolarized macrophages (M0) activated to a pro‐inflammatory type.^16^, ^17^ As ESHP should not exceed 6 h,^18^ only M1‐ and M0‐macrophages can be expected, not the anti‐inflammatory M2 population that infiltrates the heart only after some days. Targeting the immune response in DCD heart transplantation^19^ and reducing inflammatory cytokines and macrophages (M1) in cardiac IRI, have been proposed to improve post‐ischaemic recovery.^20^

During ESHP, EV secreted by macrophages could affect graft quality. Indeed, M1‐derived EV, but not M0‐derived EV, have been reported to induce increased myocyte death.^13^ Metabolic modulation could also be of interest as EV have been demonstrated to upregulate glucose uptake and glycolysis,^21^ and stimulation of glucose uptake, glycolysis, glucose oxidation and the coupling of glycolysis to glucose oxidation are recognized to reduce IRI.^22^

However, the precise effects and mechanism(s) by which EV may affect post‐ischaemic cardiac recovery and glucose metabolism remain to be determined. Key regulators that stimulate glucose uptake and glycolysis are AMP‐activated protein kinase (AMPK)^23^ and serine/threonine kinase (Akt),^24^ along with their common downstream target, TBC1 domain family member 4 (AS‐160).^25^ Activation of Akt may be reflected by the phosphorylation of its downstream target GSK3β, while AMPK activity can be detected by its downstream target acetyl‐CoA carboxylase (ACC). Importantly, EV from various cell types have been demonstrated to promote AMPK and Akt pathways,^7^, ^17^, ^26^ Upon uptake, EV can induce the metabolic phenotype of secreting (parent) cells in target cells.^7^ Therefore, EV present during ESHP may transmit the macrophage phenotype to the recipient cells and alter the glucose metabolism.

We hypothesized that intravascular administration of EV from M0‐ or M1‐derived macrophages during early reperfusion affects post‐ischaemic cardiac recovery, potentially by modifying glucose metabolism, in an isolated rat model of DCD.

## 2 METHODS

### 2.1 Ethics statement

All experiments were approved by the Swiss animal welfare authorities and ethics committee for animal experimentation, Berne, Switzerland and conducted under compliance of the European convention for animal care. All measures were taken to reduce animal suffering.

### 2.2 Study design

Extracellular vesicles (EV) were isolated from blood plasma of healthy volunteers who agreed to donate blood for research purposes, as previously described.^13^

**FIGURE 1:**
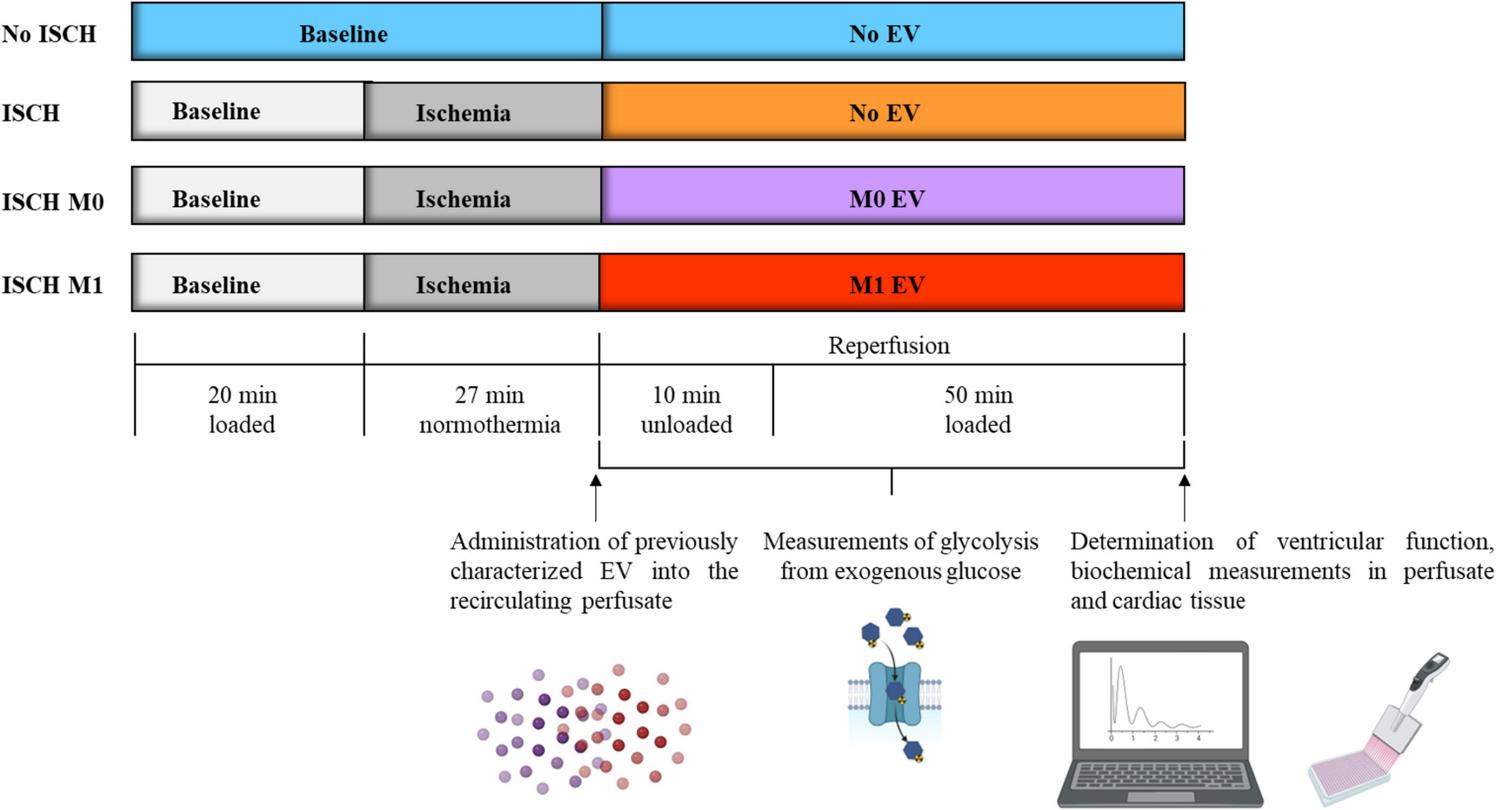
Experimental protocol. Four, parallel‐arm experimental groups (No ISCH, ISCH, ISCH M0 and ISCH M1) were compared. Hearts were perfused for an aerobic baseline period, followed by further aerobic perfusion (No ISCH), or warm (37°C), global ischaemia and reperfusion. During baseline, hearts were perfused with modified Krebs–Henseleit buffer containing glucose (10 mM), palmitate (1.2 mM) and lactate (1 mM) to simulate DCD conditions. During ischaemia, hearts were immersed in 37°C energy substrate‐free buffer. During reperfusion, either no EV, or EV from M0‐macrophages or M1‐macrophages were added to the perfusate, which consisted of modified Krebs–Henseleit buffer containing ^3^H‐glucose for the measurement of exogenous glucose metabolism, without added fatty acids or lactate. For the first 10 min of reperfusion, hearts were perfused in an unloaded mode with a limited volume of recirculating reperfusion buffer and a corresponding custom‐made oxygenator. For the remaining 50 min of reperfusion, standard volumes and oxygenation were used, and the left ventricle was loaded. Samples of perfusate were collected at multiple timepoints to assess metabolic and biochemical parameters, while cardiac tissue was harvested at the end of reperfusion. DCD, donation after circulatory death; EV, extracellular vesicles; ISCH M0, ischaemia with M0‐derived EV; ISCH M1, ischaemia with M1‐derived EV; No ISCH, no ischaemia; No EV ISCH, ischaemia without EV.

Male Wistar rats (Rattus norvegicus, Janvier Labs, Le Genest‐Saint‐Isle, France), aged 10–11 weeks, were used to model young, adult DCD donors. Block randomization was used to assign rats to one of four, parallel‐arm experimental groups: no ischaemia, no EV (No ISCH), ischaemia without EV (ISCH), ischaemia with M0‐derived EV (ISCH M0) and M1‐derived EV (ISCH M1) as shown in Figure 1.

### 2.3 Experimental model

We have previously established the isolated rat heart model of DCD in our laboratory with a 20‐min baseline period to allow for heart stabilization after explantation, followed by a 27‐min period of warm, global ischaemia.^27^ During the baseline perfusion, DCD conditions are modelled in the isolated heart with the addition of supraphysiologic levels of palmitate and lactate prior to warm, global ischaemia.^28^, ^29^ The ischaemic duration of 27 min provides an intermediate cardiac recovery, which is of particular importance to achieve measurable effects (either positive or negative) for interventions applied at the time of reperfusion, and it also corresponds well with clinical warm ischaemic durations, reported as approximately 23–29 min.^1^ The ex‐vivo perfusion model was chosen to limit the effects of potentially confounding EV released from multiple tissues in the donor during simulation of withdrawal of life support.

### 2.4 EV isolation and characterization

Briefly, monocytes were isolated from human buffy coats and cultivated in fetal bovine serum for the M0‐macrophages or in polarization media that contained interferon‐gamma and lipopolysaccharide for the M1‐polarization. We were especially interested in investigating the short‐term effects of pro‐inflammatory M1 and unpolarized EV on the isolated heart, as these types of macrophages are expected to be present at our timepoints of interest for reperfusion, which currently takes place for several hours following donor death. This is in contrast to M2 macrophages, which are present during the resolution and repair phase following an ischaemic event that starts only days after an ischaemic incident; thus few M2 macrophages are expected to be present during ESHP. The induced polarization was confirmed with PCR (Figure S1). EV were then isolated with ultracentrifugation, quantified with the nanoparticle tracking analysis (NTA) and characterized using western blot (Figure S2).

### 2.5 Experimental protocol

Rats were anaesthetised via intraperitoneal injection of 100 mg/kg ketamine (Narkatan, Vetoquinol AG, Bern, Switzerland) and 10 mg/kg xylazine (Xylapan, Vetoquinol AG, Bern, Switzerland). After disappearance of the pedal reflex, hearts were explanted and ESHP was initiated according to the protocol (Figure 1). Briefly, hearts underwent 20 min baseline period followed by either 27 min of warm, global ischaemia (ISCH) or continued aerobic perfusion (non‐ischaemic control; No ISCH). Hearts were then reperfused for 1 h to assess recovery. At the beginning of reperfusion, no EV or EV from either M0‐ or M1‐macrophages (ISCH M0; ISH M1) were administered and recirculated throughout the entire reperfusion.

During reperfusion, recovery or ventricular function was monitored continuously using a micro‐tip pressure catheter inserted into the left ventricle (detailed description in the supplementary methods). Perfusate samples were taken to assess the glycolysis (0, 20, 40 and 60 min). Cell death marker release into the perfusate was measured at 0, 20 and 60 min of reperfusion. As previously reported,^30^ these levels generally increase gradually during reperfusion and are most pronounced at 60 min reperfusion. We therefore chose the 60 min timepoint as the most representative value for the entire reperfusion duration. Similarly, with this model, cytochrome‐C levels in perfusate increase during reperfusion and are most pronounced at 60 min,^31^ which is why cytochrome‐C levels at this timepoint were considered to be most representative. Oxygen consumption and lactate concentrations were reported at the 60‐min reperfusion timepoint in order to correspond with the times at which ventricular recovery and cell death markers were reported.

Detailed heart perfusion methods and biochemical measurements are described in Supplementary Material.

### 2.6 Statistical analysis

Values are reported as median and interquartile range unless otherwise stated. Statistical calculations were performed with GraphPad Prism version 9 (GraphPad Software, Inc., La Jolla, CA). Individual values identified as outliers with the Tukey test were excluded from the study. Differences among experimental groups were identified with the Kruskal–Wallis, and when significant, pairwise comparisons were made with the Mann–Whitney *U*‐test. Cell death marker release was correlated to functional recovery using Spearman correlations. Rates of glycolysis were compared using linear regressions. *P* values were adjusted for multiple comparisons (modified, sequential and rejective Bonferroni procedure).^32^ Statistical significance is indicated when corrected *p* < 0.05.

## 3 RESULTS

### 3.1 Macrophage polarization, EV isolation and characterization

Macrophages were isolated from healthy human buffy coats. Detailed results of macrophage isolation, cultivation and polarization, as well as EV isolation and characterization are presented in the Section S1.

### 3.2 Baseline characteristics

Baseline characteristics are shown in the supplementary results (Table S4). A total of 25 rats was included in the study, with a sample size of 6–7 per experimental group. No difference among experimental groups was observed, except for cardiac output and coronary flow. Cardiac output was significantly higher in No ISCH than in ISCH M0 and ISCH M1, and coronary flow was significantly higher in the No ISCH group compared to ISCH M1 (*p* < 0.05, for all).

### 3.3 Ventricular recovery and cell death

**FIGURE 2:**
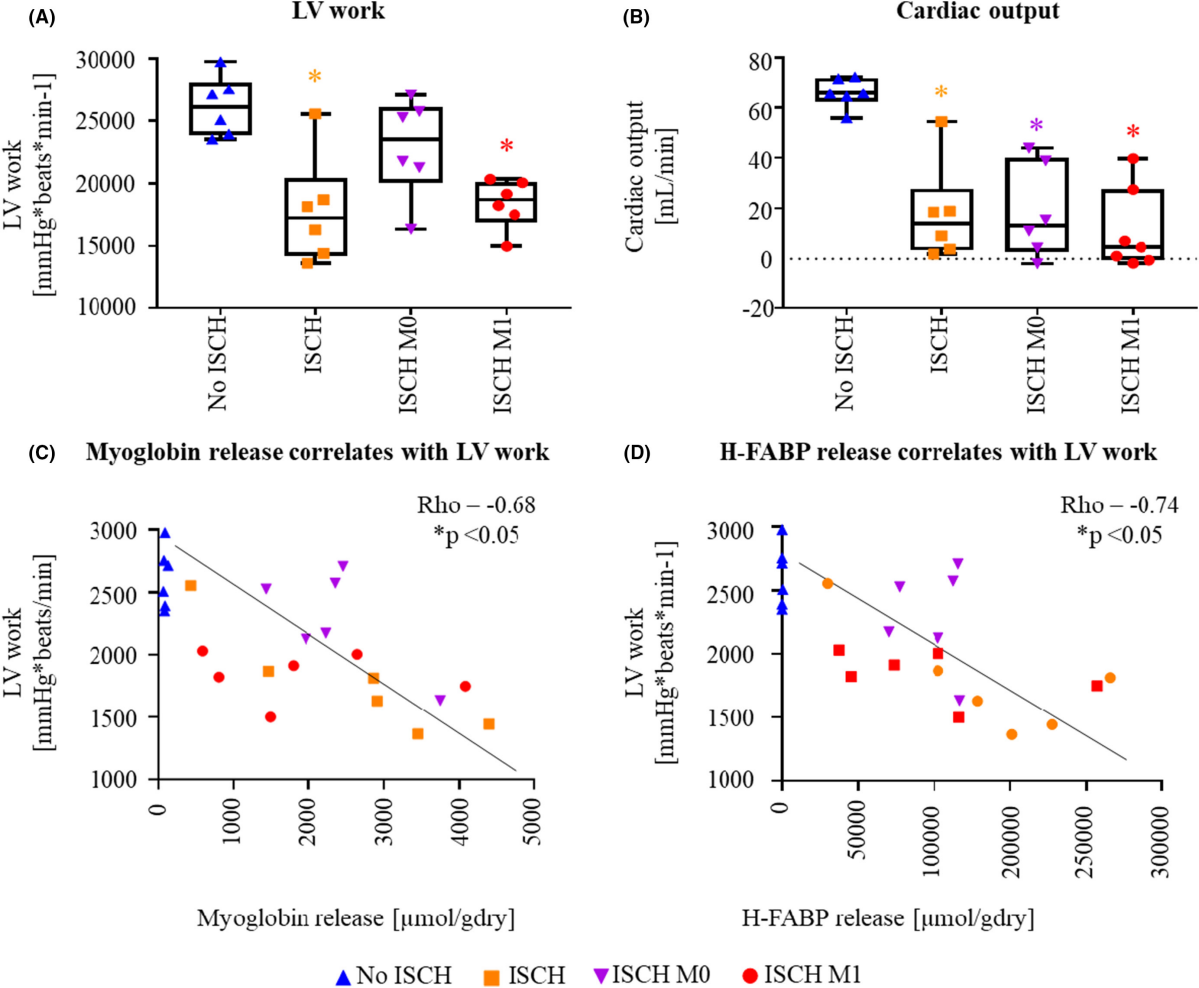
Functional recovery and cell death release at 60 min reperfusion. (A) Left ventricular work (LV work), (B) cardiac output, (C) Spearman correlation of myoglobin release during reperfusion with LV work, rho = −0.68 and (D) Spearman correlation of heart‐type fatty acid binding protein (H‐FABP) release during reperfusion with LV work, rho = −0.74, *p* < 0.05 for both. ISCH: ischaemia without EV, ISCH M0: ischaemia with M0‐derived EV, ISCH M1: ischaemia with M1‐derived extracellular vesicles (EV), No ISCH: no ischaemia without EV. *p < 0.05 versus No ISCH, *n* = 6–7/group.

At 60 min reperfusion (Figure 1), left ventricular (LV) work was highest in No ISCH and ISCH M0, compared to ISCH and ISCH M1 reaching statistical significance only for No ISCH versus ISCH and ISCH M1 (*p* < 0.05 for both; Figure 2A). Cardiac output was significantly lower in the ischaemic groups compared to No ISCH (*p* < 0.05 for all; Figure 2B). Release of myoglobin and heart‐type fatty acid‐binding protein (H‐FABP) negatively correlated with LV work (*p* < 0.05 for both; Figure 2C,D).

Overall, we observed a significant decrease in left ventricular work in the ISCH and ISCH M1 hearts, but not ISCH M0, compared to No ISCH. Cell death marker release at 60 min reperfusion significantly correlated with functional recovery. Thus, macrophage‐derived EV administration at reperfusion differentially affects post‐ischaemic cardiac recovery depending on parent cell polarization status.

### 3.4 Glucose metabolism

**FIGURE 3:**
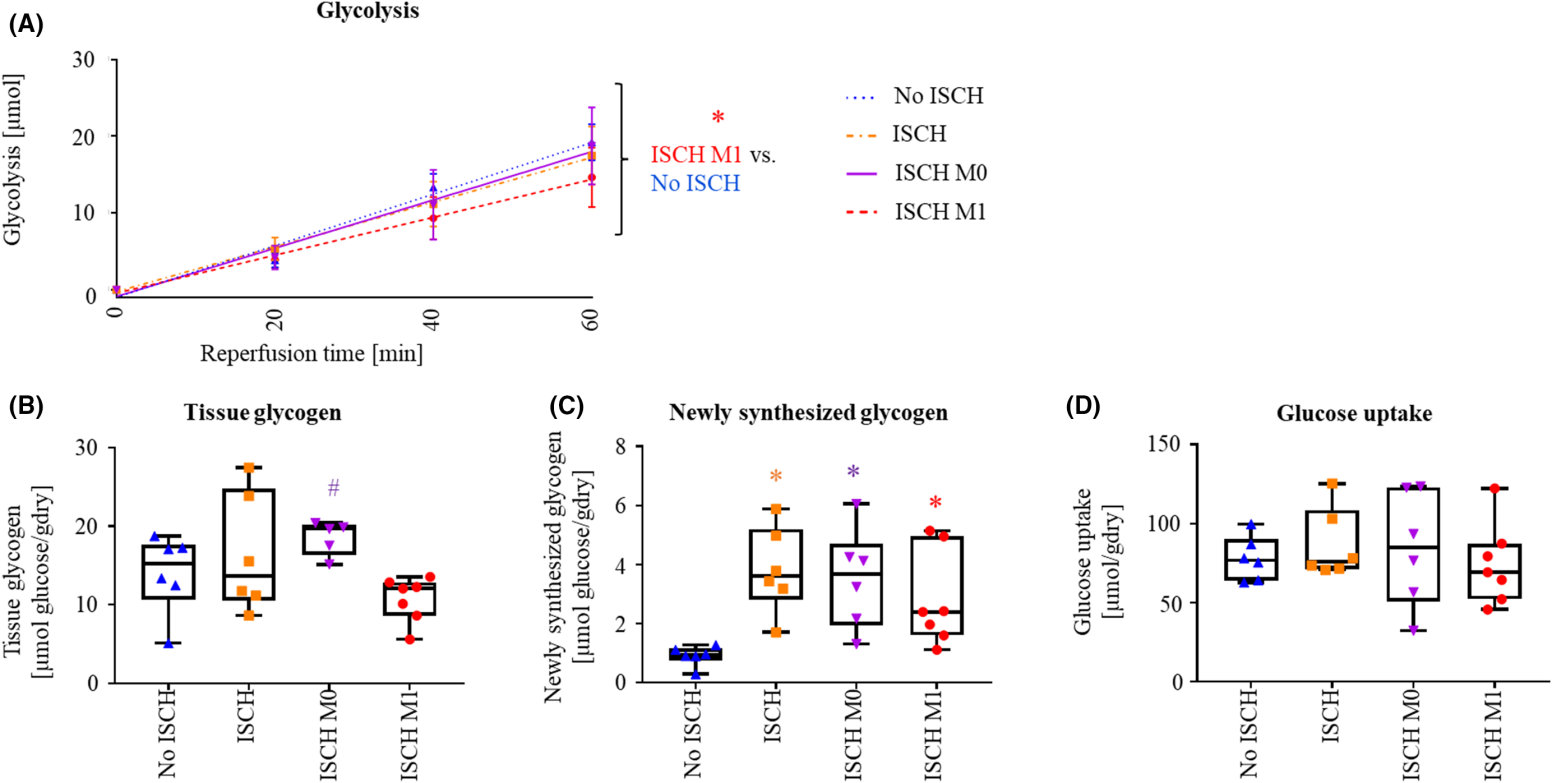
Glucose metabolism during reperfusion. (A) Glycolysis from exogenous glucose (mean and standard deviation are reported). (B) Total tissue glycogen at 60 min reperfusion. (C) Glycogen synthesized during reperfusion. (D) Calculated glucose uptake. ISCH, ischaemia without extracellular vesicles (EV); ISCH M0, ischaemia with M0‐derived EV; ISCH M1, ischaemia with M1‐derived EV; No ISCH, no ischaemia without EV. *p < 0.05 versus No ISCH, ^#^ *p* < 0.05 versus ISCH M1, *n* = 4–7/group.

Over the 60‐min reperfusion period (Figure 1), rates of glycolysis tended to be lower in ISCH M1 compared to all other groups, reaching statistical significance for ISCH M1 compared to No ISCH (*p* < 0.05; Figure 3A). Tissue glycogen at 60 min reperfusion was significantly lower in ISCH M1, compared to ISCH M0 (*p* < 0.05; Figure 3B). Glycogen synthesized during reperfusion was significantly higher in the ischaemic groups compared to No ISCH (*p* < 0.05 for all; Figure 3C). Calculated glucose uptake during reperfusion was similar among experimental groups (Figure 3D).

In summary, ISCH M1 demonstrated a tendency for lower rates of glycolysis from exogenous glucose, compared to No ISCH, ISCH and ISCH M0. Furthermore, the glycogenolysis was increased in ISCH M1 compared to ISCH M0. Taken together, it appears that EV from M1‐macrophages promote use of endogenous glucose (glycogenolysis), while EV from M0‐macrophages does not. Thus macrophage‐derived EV administration at reperfusion differentially affects cardiac post‐ischaemic glucose metabolism according to parent cell polarization status.

### 3.5 Oxidative metabolism and adenine nucleotides

**FIGURE 4:**
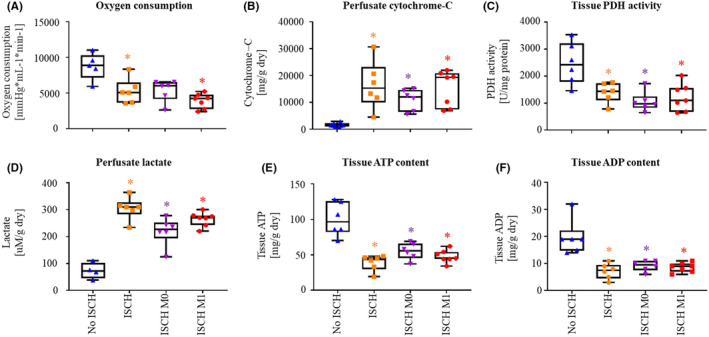
Metabolic recovery at 60 min reperfusion. (A) Oxygen consumption. (B) Cytochrome‐C release. (C) Tissue pyruvate dehydrogenase (PDH) activity. (D) Lactate release. (E) Tissue ATP content. (F) Tissue ADP content. ISCH, ischaemia without extracellular vesicles (EV); ISCH M0, ischaemia with M0‐derived EV; ISCH M1, ischaemia with M1‐derived EV; No ISCH, no ischaemia without EV. *p < 0.05 versus No ISCH, *n* = 4–7/group.

At 60 min reperfusion (Figure 1), oxygen consumption followed the same pattern as LV work, with No ISCH and ISCH M0 appearing higher than ISCH and ISCH M1, but only reaching statistical significance for No ISCH versus ISCH and ISCH M1 (*p* < 0.05 for both; Figure 4A). Mitochondrial damage, measured by the release of cytochrome‐C over 60 min reperfusion, was significantly increased in all ischaemic groups compared to No ISCH (*p* < 0.05 for all; Figure 4B), with a tendency for reduced cytochrome‐C release in ISCH M0 compared to ISCH M1. Tissue pyruvate dehydrogenase (PDH) activity was significantly decreased in ischaemic groups compared to No ISCH (*p* < 0.05 for all; Figure 4C). Compared to LV work and oxygen consumption, an inverse profile was observed for perfusate lactate (Figure 4D). Tissue ATP and ADP were significantly lower in ischaemic groups compared to No ISCH (*p* < 0.05 for all), with a non‐significant tendency for higher levels in ISCH M0 versus ISCH and ISCH M1 (Figure 4E,F). Oxidative stress was not different among ischaemic groups, nor was it correlated with functional outcomes (data not shown).

In summary, our data indicate that oxidative metabolism is reduced in ISCH M1 compared to ISCH M0, as demonstrated by trends for reduced oxygen consumption and tissue ATP levels, in parallel with increased cytochrome‐C and lactate release.

### 3.6 Key signalling molecules in glucose metabolism

**FIGURE 5:**
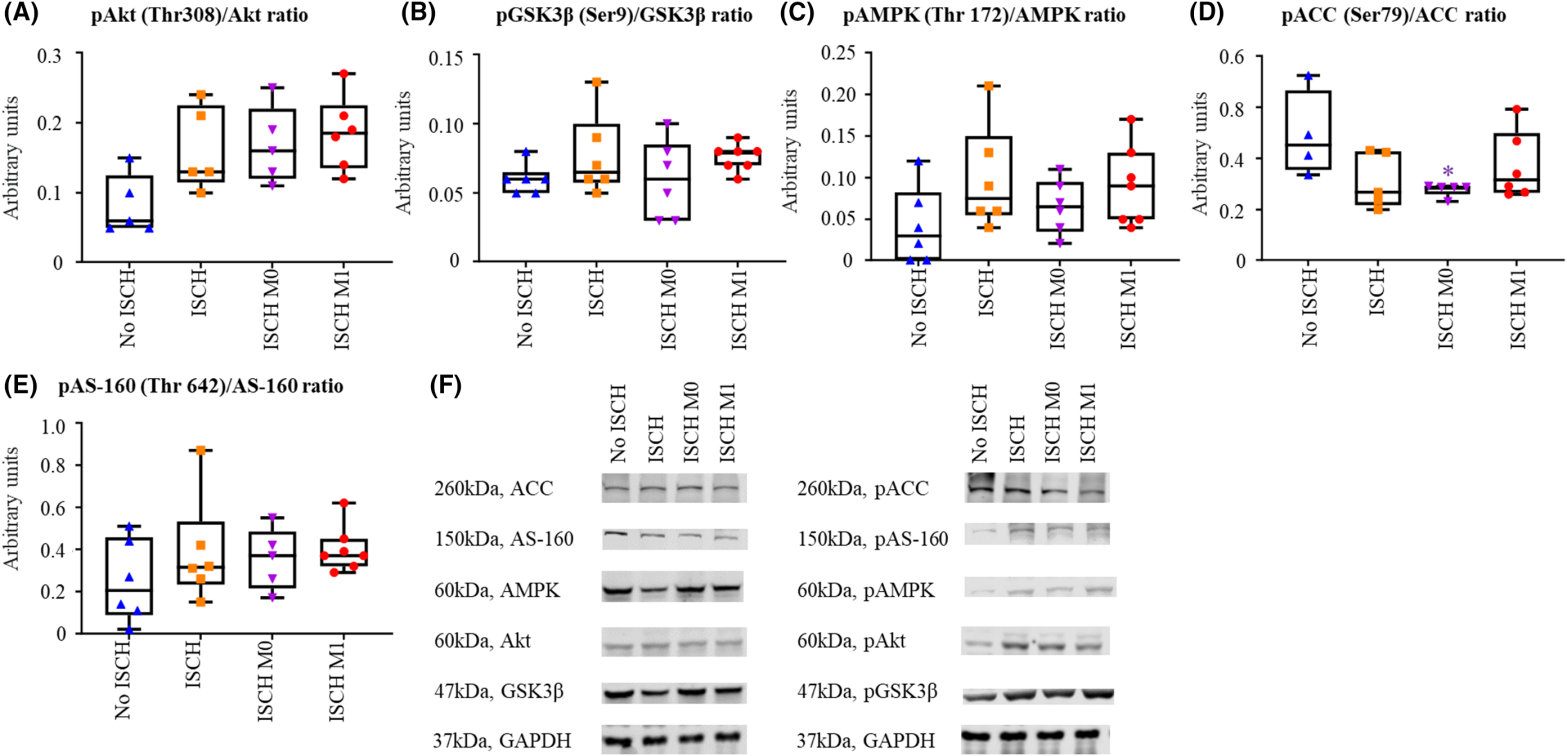
Phosphorylation of key signalling molecules at 60 min reperfusion. (A) Serine/threonine kinase (Akt). (B) glycogen synthase kinase 3β (GSK3β). (C) AMP‐activated protein kinase (AMPK). (D) Acetyl‐CoA carboxylase (ACC). (E) TBC1 domain family member 4 (AS‐160) and (F) representative bands for the total and phosphorylated signalling molecules as well as the loading control GAPDH. ISCH, ischaemia without EV; ISCH M0, ischaemia with M0‐derived EV; ISCH M1, ischaemia with M1‐derived EV; No ISCH, no ischaemia without EV. *p < 0.05 versus No ISCH, *n* = 6–7/group.

Phosphorylation of key signalling molecules involved in the regulation of glucose metabolism was assessed in ventricular tissue, harvested at the end of reperfusion (Figure 1) by western blot and presented in Figure 5. Uncut membranes are shown in supplementary Figure S3. Phosphorylation of Akt (Thr 308) tended to be increased in all ischaemic groups compared to No ISCH (Figure 5A), demonstrating increased activation of Akt in ischaemic groups and consistent with trends for increased phosphorylation of its downstream target GSK3β (Ser‐9; Figure 5B). AMPK phosphorylation (Thr 172) tended to be increased in ISCH and ISCH M1 hearts compared to No ISCH and ISCH M0 hearts (Figure 5C). However, this pattern of activation for AMPK was not clearly maintained for the phosphorylation of its downstream target, ACC (Ser 79; Figure 5D). Akt and AMPK kinase signalling pathways converge to phosphorylate AS160 (Thr 642), which demonstrates a tendency for higher phosphorylation in ischaemic groups compared to No ISCH, but no difference among ischaemic groups (Figure 5E).

Overall, our data indicate that ischaemia provoked the activation of Akt and AMPK pathways. However, whereas the phosphorylation of these pathways appeared to be unchanged by the administration of EV for Akt signalling, a trend for greater activation of AMPK signalling was observed in ISCH M1 versus ISCH M0 hearts.

### 3.7 Gene expression

**FIGURE 6:**
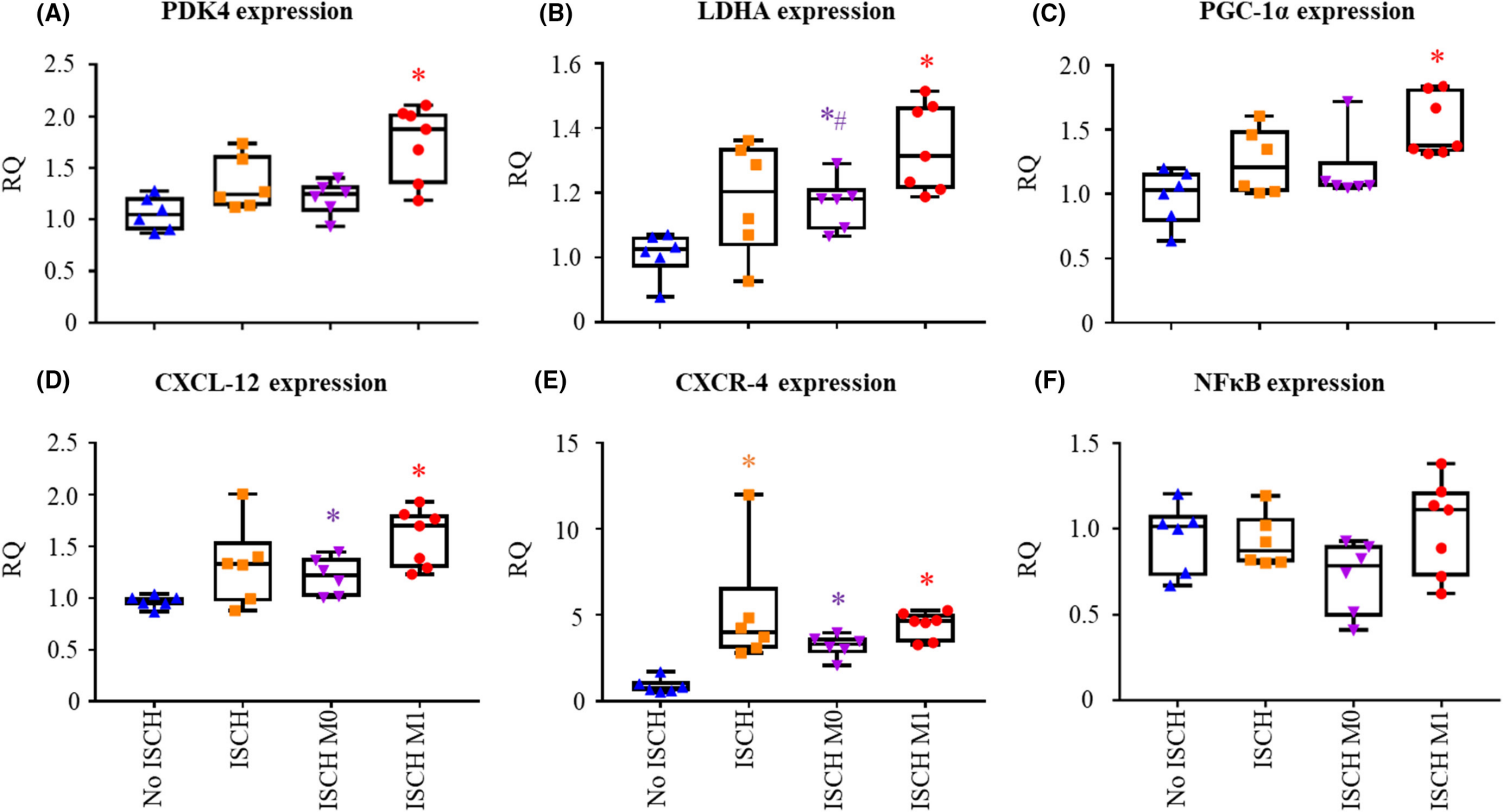
Expression of metabolic and inflammatory genes at 60 min reperfusion. (A) Pyruvate dehydrogenase kinase 4 (PDK4). (B) Lactate dehydrogenase A (LDHA). (C) Peroxisome proliferator activated receptor gamma coactivator‐1 alpha (PGC1‐α). (D) CXC‐motive‐chemokine 12 (CXCL‐12). (E) CXC‐motive receptor (CXRC‐4) and (F) Nuclear factor kappa beta (NFκB). Gene expression is reported as relative quantity (RQ). ISCH, ischaemia without EV; ISCH M0, ischaemia with M0‐derived EV; ISCH M1, ischaemia with M1‐derived EV; No ISCH, no ischaemia without EV. *p < 0.05 versus No ISCH, ^#^ *p* < 0.05 versus ISCH M1, *n* = 6–7/group.

Gene expression was assessed in the tissue, harvested at the end of reperfusion (Figure 1) and is presented in Figure 6. Pyruvate dehydrogenase kinase 4 (PDK4) and lactate dehydrogenase A (LDHA) expression were used to provide information about metabolic changes, specifically glycolysis–glucose oxidation uncoupling. Peroxisome proliferator‐activated receptor gamma coactivator‐1 alpha (PGC‐1α) expression is increased by cellular stress, such as ischaemia, and is involved in regulating the expression of multiple genes involved in energy metabolism.^33^ CXC‐motive‐chemokine ligand 12 (CXCL‐12) and CXC‐motive chemokine receptor (CXCR‐4) expression are increased after ischaemia and involved in multiple cellular processes including inflammation.^34^ PDK4 was significantly increased in ISCH M1 compared to No ISCH (*p* < 0.05) and tended to be higher than ISCH M0 (*p* = 0.066; Figure 6A). LDHA (Figure 6B) tended to be increased in ischaemic groups compared to No ISCH, reaching significance for ISCH M1 and ISCH M0 (*p* < 0.05). Also, LDHA expression was significantly higher in ISCH M1 compared to ISCH M0 (*p* < 0.05). Expression of PGC‐1α was significantly higher in ISCH M1 compared to No ISCH (*p* < 0.05, Figure 6C). CXCL‐12 tended to be upregulated in all ischaemic groups compared to No ISCH, reaching statistical significance for ISCH M0 and ISCH M1 (*p* < 0.05 for both, Figure 6D). CXCR‐4 was significantly upregulated in all ischaemic groups compared to No ISCH (*p* < 0.05, Figure 6D). Nuclear factor kappa beta (NFκB) tended to be reduced in ISCH M0 compared to ISCH M1 (Figure 6F). Interleukin‐6 expression was similar among groups, while expression of interleukin‐1β and tumour necrosis factor alpha mRNA was lower in ischaemic groups compared to No ISCH, but not altered between M0 and M1 (Figure S4).

Taken together, our gene expression results support metabolic and biochemical findings of reduced oxidative metabolism and mitochondrial integrity in ISCH M1 compared to ISCH M0, with corresponding increases in PDK‐4, LDHA and PGC‐1α expression. Furthermore, trends for increased pro‐inflammatory gene expression in ISCH M1 versus ISCH M0 are demonstrated by trends of increased CXCL‐12, CXCR‐4 and NFκB expression.

## 4 DISCUSSION

**FIGURE 7:**
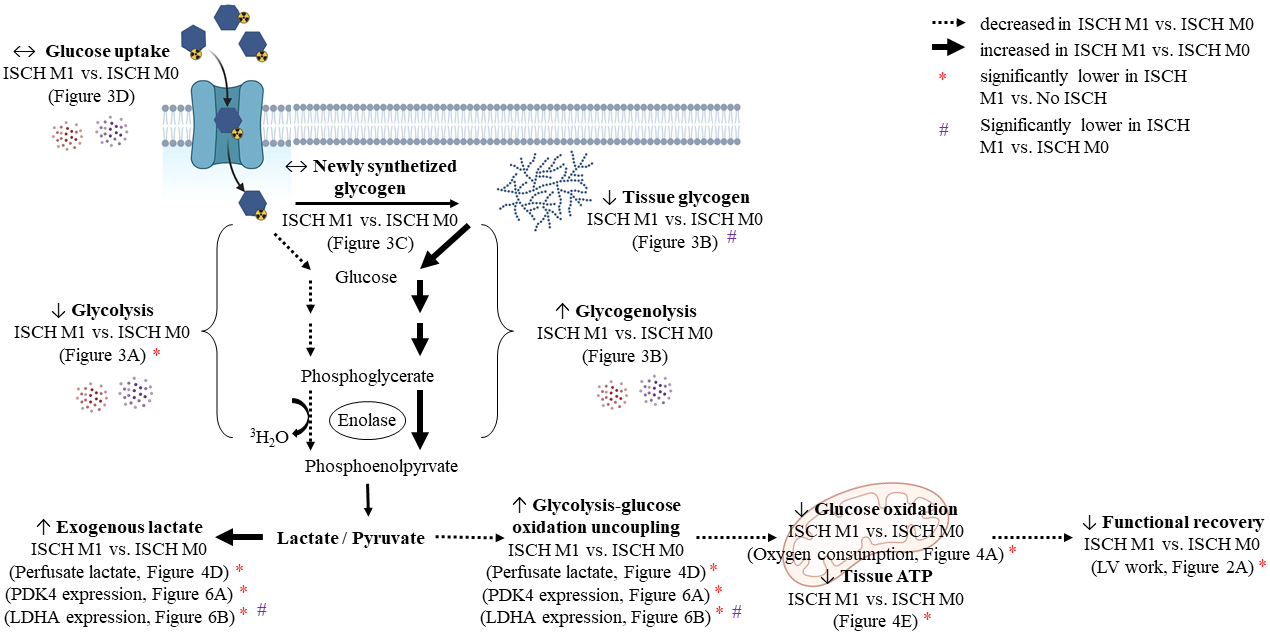
Proposed mechanism for macrophage‐derived EV effects in hearts treated during reperfusion. Glycolysis from exogenous glucose tends to be reduced in ISCH M1 compared to ISCH M0, whereas glycolysis from endogenous glucose (glycogen) tends to be increased in ISCH M1 compared to ISCH M0. Release of lactate tends to be increased in ISCH M1 versus ISCH M0, while oxygen consumption and tissue ATP tends to be decreased, indicating reduced oxidative metabolism and energy availability. Overall, the observed glycolysis–glucose oxidation uncoupling during reperfusion may contribute to lower post‐ischaemic ventricular recovery in ISCH M1 compared to ISCH M0. ISCH, ischaemia without EV; ISCH M0, ischaemia with M0‐derived EV; ISCH M1, ischaemia with M1‐derived EV; No ISCH, no ischaemia without EV.

In DCD heart transplantation, EV management, either administration of beneficial EV or removal of detrimental EV, could help to optimize graft quality. To our knowledge, this study is the first to examine and compare the effects of EV from unpolarized macrophages (M0) and pro‐inflammatory macrophages (M1) during ESHP. As expected, exposure to ischaemia and reperfusion significantly decreased ventricular function, and this remained unchanged by the administration M1‐EV, while administration of M0‐EV tended to improve ventricular function. We propose an improved coupling of glycolysis to glucose oxidation as the mechanism behind the improved recovery observed in M0 compared to M1 (Figure 7). In concert with lower functional recovery in ISCH M1 versus ISCH M0, rates of glycolysis from exogenous glucose, oxidative metabolism and tissue ATP levels tended to be lower, while rates of glycogenolysis and lactate release tended to be higher. Taken together, these findings indicate that exposure of post‐ischaemic hearts to circulating EV derived from M1 macrophages cause uncoupling of glycolysis from glucose oxidation, which may contribute to the reduced cardiac recovery. Greater glycolysis–glucose oxidation uncoupling is recognized to lead to decreased post‐ischaemic cardiac recovery and is in line with reported effects of EV transmission of metabolic changes observed in M1‐like phenotypes. Therefore, we conclude that in the context of DCD heart transplantation, circulating EV during ESHP may alter graft quality, potentially via modified energy metabolism, and thus warrant further research attention. An important corollary is that circulating EV may be targeted during ESHP in order to optimize cardioprotection.

EV that are released into the circulation by multiple cell types during donor death and remain present during reperfusion may have molecular and immunologic properties that can influence recovery of the heart. We observed tendencies for ischaemia‐induced ventricular dysfunction to be reduced with M0‐EV (compared to no EV administration) and to be greater with ISCH M1 compared to ISCH M0. Importantly, the release of cell death markers significantly correlated with functional recovery, indicating increased lethal reperfusion injury with greater ventricular function impairment. In a previous study, cell viability of neonatal rat ventricular myocytes cultured in the presence of EV from M1‐ and M0‐macrophages was decreased with M1‐EV compared to M0‐EV,^13^ which is in line with our findings.

Modified energy metabolism is one mechanism by which EV may alter functional recovery of the heart. We observed the same pattern across experimental groups for recovery of LV work and oxygen consumption, highest in No ISCH and ISCH M0, compared to ISCH and ISCH M1 (reaching statistical significance only for No ISCH vs. ISCH and ISCH M1); while in ISCH M1 compared to ISCH M0 hearts, cytochrome‐C release, rates of glycogenolysis, and lactate release tended to be higher and tissue ATP levels tended to be lower. These findings support the concept that increases in oxidative metabolism contribute to improved ventricular function and are in line with previous reports, indicating that a metabolic shift away from glucose oxidation in post‐ischaemic hearts is associated with lower functional recovery.^22^, ^35^ Importantly, it is recognized that activated macrophages release EV containing M1‐polarized RNA^36^ that could induce an M1‐like phenotype in recipient cells. In M1‐macrophages, glycogen use (glycogenolysis) is stimulated.^37^ Therefore, M1‐EV, mimicking their parent cell,^7^ may also stimulate a similar metabolic switch in DCD hearts.

The mechanism behind the observed changes in glucose metabolism (and cardiac function) between ISCH M1 and ISCH M0 hearts could be through altered activation of key regulatory enzymes. As no difference among ischaemic groups was observed for the Akt signalling pathway, metabolic changes are not likely to result from Akt. In contrast, AMPK signalling tended to be increased in ISCH M1 versus ISCH M0 hearts. Increased AMPK activity promotes glycogenolysis and could therefore explain our findings of greater glycogen use in ISCH M1 versus ISCH M0.^38^ Although the precise mechanisms for EV‐induced changes in post‐ischaemic cardiac glucose metabolism remain to be fully characterized, they are consistent with the concept that the parent cell‐specific phenotype is transmitted.

Administration of EV during reperfusion differentially altered the expression of metabolic genes according to macrophage (parent cell) polarization. PDK4 expression tended to be increased in ISCH M1 versus ISCH M0. Greater PDK4 expression suggests decreased PDH activity and glycolysis–glucose oxidation uncoupling, which is in agreement with the trends of decreased oxygen consumption and increased lactate release in ISCH M1 versus ISCH M0. This would reduce the protective effect of upregulated glucose metabolism after ischaemia^35^ and is in line with lower cardiac function recovery observed in ISCH M1 versus ISCH M0. Although measured PDH activity was significantly reduced with ischaemia, no difference was observed among ischaemic groups. Nonetheless, measured PDH activity does not necessarily reflect in‐situ activity, as PDH is regulated by multiple allosteric mediators that may be altered upon isolation. Consistent with glycolysis–glucose oxidation uncoupling^39^ and greater lactate release in ISCH M1 versus ISCH M0, LDHA expression was also increased. Furthermore, PGC‐1α transcription is increased with greater lactate production^40^ as well as by activated AMPK,^33^ which is line with our findings, and supports the concept of reduced oxidative metabolism in ISCH M1 versus ISCH M0. PGC‐1α expression is also stimulated by IRI,^33^ indicating a greater ischaemic dearrangements in mitochondria of ISCH M1 versus ISCH M0, which fits with our findings of a tendency for lower oxygen consumption and greater cytochrome‐C release.

EV from differentially polarized macrophages could be expected to have different cargos and induce different gene transcription patterns for chemokines and inflammatory responses. EV from M1 macrophages have been shown to trigger inflammation,^41^ while M0‐ and M2‐macrophages were shown to enhance vascularization of tissue scaffolds.^42^, ^43^ Lastly, as M2 macrophages are recognized for their tissue and extra cellular matrix remodelling capacities,^44^ addition of M2‐EV may also promote a remodelling effect. EV of pro‐inflammatory macrophages have been reported to induce heart dysfunction by activating the NFκB pathway.^13^ These findings are in line with our observed tendency for increased NFκB expression in ISCH M1 versus ISCH M0. Even though M1‐EV are thought to have pro‐inflammatory cytokine mRNA as cargo, the suggested mechanism of action is activation of NFκB via TLR4,^13^ rather than the cytokines themselves; however, no differences in the expression of downstream inflammatory cytokines were observed under the conditions used in our study. CXCL‐12 expression is increased after ischaemia and binds to CXCR‐4 to attract inflammatory cells.^34^ Our data show tendencies for increased expression CXCL‐12 and CXCR‐4 in ischaemic groups compared to No ISCH as well as in ISCH M1 versus ISCH M0. Overall, these results indicate that stimulation of pro‐inflammatory pathways may be increased ISCH M1 versus ISCH M0.

Several limitations of this study should be noted. Only male rats were used, thereby limiting the generalization to female hearts. In addition, the reperfusion buffer used in this study did not contain blood, whereas in human heart transplantation the ESHP perfusate comprises both donor blood and physiological buffer.^45^ Therefore, in order to extend these findings to different populations and the clinical setting, further studies are required to investigate the role of EV during ESHP.

## 5 CONCLUSIONS

Intravascular administration of EV during reperfusion altered post‐ischaemic cardiac recovery in an isolated rat heart model of DCD. Treatment with EV derived from M1‐polarized macrophages led to reduced ventricular function, potentially as a result of greater glycolysis–glucose uncoupling, reduced oxidative metabolism and lower ATP levels, compared with EV derived from M0‐polarized macrophages. The metabolic changes reported in hearts exposed to EV derived from M1‐polarized macrophages are in line both with mechanisms recognized to limit post‐ischaemic cardiac recovery and with the changes observed in M1 macrophages themselves, consistent with EV transmission of parent phenotypes. Approaches that target circulating EV during ESHP may help to improve clinical DCD protocols and optimize graft quality by providing cardioprotection.

### 5.1 Clinical perspective

Therapeutic EV targeting during ESHP could be beneficial for optimizing DCD cardiac graft quality. ESHP offers a possibility to administer cardioprotective compounds,^45^ and this is of particular interest for EV. The shortcomings of low retention, short‐lived therapeutic effects as well as off‐target delivery wiht EV^46^ are less of a concern in ESHP, as in an isolated organ the off‐target delivery is limited and more EV can be administered to increase retention. As ESHP duration is on average 3.5 h^47^ and should not exceed 6 h,^18^ the short life of EV is not a concern. EV‐targeting during ESHP^6^ could be applied by intravascular administration of cardioprotective EV, or the secretion of harmful EV could inhibited with the compound GW4869.^48^

## AUTHOR CONTRIBUTIONS

**Selianne Graf:** Data curation; formal analysis; investigation; project administration; writing – original draft. **Vanessa Biemmi:** Investigation; methodology; writing – review and editing. **Maria Arnold:** Conceptualization; formal analysis; investigation; writing – review and editing. **Adrian Segiser:** Conceptualization; investigation; writing – review and editing. **Anja Müller:** Formal analysis; investigation; writing – review and editing. **Natalia Méndez‐Carmona:** Data curation; formal analysis; writing – review and editing. **Manuel Egle:** Data curation; formal analysis; writing – review and editing. **Matthias Siepe:** Funding acquisition; ; validation; writing – review and editing. **Lucio Barile:** Conceptualization; data curation; funding acquisition; methodology; supervision ; validation; writing – review and editing. **Sarah Longnus:** Conceptualization; data curation; formal analysis ; funding acquisition; methodology; ; supervision; validation; writing – original draft; writing – review and editing.

## FUNDING INFORMATION

This study was funded by a Swiss National Science Foundation COST grant (IZCOZ0_182948/1).

## CONFLICT OF INTEREST STATEMENT

None of the authors has a financial relationship or other conflicts of interest to declare, except NMC who works for Roche Switzerland; however, the employment only started after completion of the work in the research group.

## Supporting information


Appendix S1:

## Data Availability

The data that support the findings of this study are available from the corresponding author upon reasonable request.
