# Supplementary material for: Macrophage‐derived extracellular vesicles alter cardiac recovery and metabolism in a rat heart model of donation after circulatory death
Source: J Cell Mol Med. 2024 Apr 23;28(8):e18281. doi: 10.1111/jcmm.18281 (PMC11037406; doi:10.1111/jcmm.18281)
Supplement: Supplementary file 1 — Appendix S1: [file JCMM-28-e18281-s001.docx]

**Macrophage-derived extracellular vesicles alter cardiac recovery and metabolism in a rat heart model of DCD**

**Contents**

[**Supplementary methods:** 3](#_Toc158494249)

[**Macrophage polarization** 3](#_Toc158494250)

[**EV isolation and characterisation** 4](#_Toc158494251)

[**Isolated rat heart perfusion** 5](#_Toc158494252)

[**Dry heart weight** 5](#_Toc158494253)

[**Cytochrome-C, myoglobin, heart-type fatty acid binding protein and lactate** 5](#_Toc158494254)

[**Glycolysis** 6](#_Toc158494255)

[**Glycogen content** 6](#_Toc158494256)

[**Newly synthesized glycogen** 6](#_Toc158494257)

[**Glucose uptake** 6](#_Toc158494258)

[**Cardiac oxygen consumption** 7](#_Toc158494259)

[**Tissue pyruvate dehydrogenase activity** 7](#_Toc158494260)

[**Tissue ATP/ADP content** 7](#_Toc158494261)

[**Protein carbonylation (oxidative stress)** 7](#_Toc158494262)

[**Key signaling molecules in glucose metabolism** 7](#_Toc158494263)

[**Gene expression** 8](#_Toc158494264)

[**Supplementary results** 9](#_Toc158494265)

[**Macrophage polarization** 9](#_Toc158494266)

[**EV characterisation** 10](#_Toc158494267)

[**Baseline characteristics** 11](#_Toc158494268)

[**Key signaling molecules in glucose metabolism** 12](#_Toc158494269)

[**Gene expression** 14](#_Toc158494270)

[**References** 14](#_Toc158494271)

# **Supplementary methods:**

## **Macrophage polarization**

Peripheral blood mononuclear cells were isolated from buffy coats of 3 healthy donors using the ficoll gradient technique. These cells were then seeded in tissue culture plates (Corning Constar, New York, USA) in RPMI 1640 medium (Gibco, Carlsbad, USA), supplemented with 10% FBS (Gibco) and 10 ng/ml M-CSF (Prospect, London, UK). 10 days after plating, adherent mature macrophages (M0) were polarized toward a pro-inflammatory M1 profile using serum-free RPMI, 10 ng/ml M-CSF, 100 ng/ml TNF-α (Prospect, London, UK), and 100 ng/ml INF-γ (Prospect, London, UK). 24h after polarization, M0 and M1 macrophages were collected and total RNA extracted with TRI-Reagent (Sigma Aldrich, St. Louis, USA) and chloroform following manufacturers’ instructions. 500ng of total RNA was reverse transcribed using GoScriptTM Reverse Transcription System (Promega, Madison, USA) following manufacturers’ instructions. Cell polarization was assessed by real-time PCR using the SYBR Green labelling protocol (BioRad, Hercules, USA) following the manufacturers’ instructions. The success of M1 polarization was assessed through real-time PCR, examining the upregulation of inducible nitric oxide synthase (INOS), indoleamine 2,3-dioxygenase (IDO), tumor necrosis factor alpha (TNF-α), C-C motif chemokine receptor 2 (CCR-2) and C-C motif chemokine ligand 11 (CCL-11; Table S1). Expression profiles of M0 and M1-macrophages were compared using a ratio paired t-test, with Bonferroni correction and reported as significant if p <0.05.

| **INOS** | F: CTC TCG GCC ACC TTT GAT GA  R: TGT TCT TCA CTG TGG GGC TTG |
| --- | --- |
| **IDO** | F: GGC CAG CTT CGA GAA AGA GT  R: TTG GCA AGA CCT TAC GGA CA |
| **TNF-α** | F: CCT CTC TCT AAT CAG CCC TCT G  R: GAG GAC CTG GGA GTA GAT GAG |
| **CCR-2** | F: TCAACTGGACCAAGCCACG  R: AAGAGCTATGTGAAAAAGGCTTCT G |
| **CCL-11** | F: GAC GCT GTC TTT GCA TAG GC  R: GGA TTT AGG CAT CGT TGT CCT TT |

Table S1 Sequence of forward (F) and reverse (R) primers for verification of macrophage polarization. Inducible nitric oxide synthase (INOS), indoleamine 2,3-dioxygenase (IDO), tumor necrosis factor alpha (TNF-α), C-C motif chemokine receptor 2 (CCR-2), C-C motif chemokine ligand 11 (CCL-11)

## **EV isolation and characterisation**

Four days after polarization, cell culture media were collected, and a series of centrifugation steps (3000g for 20min at 10°C and 10000g for 30min at 10°C) were performed to eliminate cellular debris. Supernatants underwent ultracentrifugation at 100000g for 4h at 10°C using a Beckmann Optima Max-TL ultracentrifuge. Pellets containing extracellular vesicles (EV) were resuspended in PBS (pH 7.4) and stored for further analysis. Prior to freezing, EV characterization was performed by nanoparticle tracking analysis (NTA). A 1:1000 dilution of each EV preparation was analyzed by particle metrix, and the concentration of EV was quantified based on three different acquisitions.

Total protein extractions from vesicles were obtained using RIPA buffer (Sigma Aldrich, St. Louis, USA) supplemented with proteases (#S8830-20TAB, Sigma Aldrich, St. Louis, USA) and phosphatase inhibitors (#HY-K0022 and #HY-K0023, Medchem Express, Monmouth Junction, USA). The lysate protein concentrations were measured using the QuantiproTM BCA assay (#QPBCA, Sigma Aldrich, St. Louis, USA) following the manufacturers’ instructions. Proteins were denatured by boiling samples at 100°C for 10min with Laemmli SDS sample buffer 6X (#J61337, Alfa Aesar, Ward Hill, USA). Subsequently, proteins were separated on a 4-20% Mini-PROTEAN®TGX precast gel (Biorad, Hercules, USA) and transferred with the semi-dry technique onto PVDF mini membranes (Biorad, Hercules, USA). EV characterization was performed by examining the expression of specific markers, including Alix (#ab186429, Abcam, Cambridge, UK), TSG101 (#ab12501, Abcam, Cambridge, UK), Syntenin (#ab133267, Abcam) and CD63 (provided by Popovic *et. al* (2018)^2^). EV were aliquoted at 1.5x10^10^ EV per tube, and stored at -80^◦^C until use.

## **Isolated rat heart perfusion**

Isolated hearts were perfused in baseline with modified Krebs-Henseleit buffer simulating DCD conditions (118mM NaCl, 4.7mM KCl, 1.2mM KH_2_PO_4_, 1.25mM CaCl_2_ 2H_2_O, 1.2mM MgSO_4_ 7H_2_O, 25mM NaHCO_3_ and 11mM glucose, supplemented with 3% bovine serum albumin, 1.2mM palmitate and 1mM lactate, oxygenated with 95% O_2_ / 5% CO_2_).

The reperfusion buffer consisted of modified Krebs-Henseleit buffer without albumin, palmitate and lactate and either with or without 1.5x10^10^ EV from M0 or M1 macrophages. 1.5x10^10^ EV corresponds to the amount of EV in the human circulation after a severe myocardial infarction^3^.

During the aerobic perfusion periods, functional data from the left ventricle were collected with a micro-tip pressure catheter (Millar, Houston, USA). Cardiac output and coronary flow were assessed with flowmeters placed in aortic and preload lines (Transonic Systems Inc., Ithaca, USA). All functional data were recorded with the PowerLab data acquisition system (ADInstruments, Sydney, Australia).

At the end of the reperfusion, hearts were snap frozen in liquid nitrogen and stored at -80^◦^C until further analysis. For all tissue measurements, hearts were powdered with a mortar and pestle cooled with liquid nitrogen.

## **Dry heart weight**

Powdered heart tissue samples were weighed and then dried at 60^◦^C for three days, until the weight was stable. The quotient obtained by dividing weight before (wet) and after drying (dry weight) was referred to as the wet-dry-weight ratio.

## **Cytochrome-C, myoglobin, heart-type fatty acid binding protein and lactate**

ELISA measurements of buffer samples from 0 minutes and 60 minutes reperfusion were performed for cytochrome-C (R&D Systems, Minneapolis, USA), lactate (Sigma Aldrich, St. Louis, USA), myoglobin and heart-type fatty acid binding protein (H-FABP) (both: Life Diagnostics, West Chester, USA). Release of factors was calculated as follows:

C = concentration in the recirculating buffer

HW_dry_ = dry heart weight

V = volume of recirculating buffer

60, 0 = 60 and 0 minutes reperfusion time points

**______________________**

C(60) * V_60_ - C(0) * V_0_

HW_dry_

## **Glycolysis**

Tritiated glucose ([5-^3^H] Glucose; PerkinElmer, Waltham, USA) was added to the reperfusion buffer as previously described^4^ and rates of glycolysis determined by the generation of ^3^H_2_O. To do so, glucose was separated from the buffer sample using anion-exchange columns (Dowex chloride form 200-400 mesh, Sigma, Buchs, Switzerland) and measured with Liquid Scintillation Analyzer (TRI-CARB 2300TR, TopLab, Rickenbach, Switzerland)^5,6^. Perfusate samples were taken at 0, 20, 40 and 60 minutes of reperfusion and rates of glycolysis were calculated using a linear regression model.

## **Glycogen content**

Cardiac tissue glycogen content was measured as previously described, using the spectrometric glucose assay kit (Sigma Aldrich, St. Louis, USA) and normalizing by the dry weight^6,7^.

## **Newly synthesized glycogen**

Newly synthesized glycogen was calculated using tritiated glucose incorporation into total tissue glycogen at 60 minutes of reperfusion.

## **Glucose uptake**

Glucose uptake was calculated as the sum of glucose newly incorporated into tissue glycogen and exogenous glucose passing through glycolysis during reperfusion.

## **Cardiac oxygen consumption**

Oxygen partial pressure in buffer samples was determined using a Cobas b 123 blood-gas analyzer (Roche, Basel, Switzerland) and oxygen consumption at a given timepoint was calculated as:

O_2_C = oxygen consumption

CE = coronary effluent

CF = coronary flow

PL = preload line

pO_2_ = oxygen partial pressure

O_2_C = (pO_2PL_ - pO_2CE_)* CF

## **Tissue pyruvate dehydrogenase activity**

Enzyme activity was measured using an ELISA technique, as described previously^6^.

## **Tissue ATP/ADP content**

ELISA measurements of tissue extracts harvested at 60 minutes reperfusion were measured according to the manufacturers’ instructions (Abcam, Cambridge, UK).

## **Protein carbonylation (oxidative stress)**

Protein carbonylation in the heart tissue was measured with OxyBlot TM Protein Oxidation Detection kit (Merck, Millipore, Burlington,USA) according to the manufacturers’ instructions. Modifications in the kits protocol were: GAPDH was used as a primary antibody (Santa Cruz, Biotechnology, Dallas, USA), diluted in 1:1’000 PBS-Odyssey Blocking Buffer (PBS-OBB, LI-COR Biosciences, Lincoln, USA) with a goat anti-mouse secondary antibody (LI-COR Biosciences, Lincoln, USA), diluted 1:10’000 in PBS-OBB, as well as a goat anti-rabbit fluorescent secondary antibody for the carbonylated proteins ( Invitrogen, Carlsbad, USA).

## **Key signaling molecules in glucose metabolism**

Western blots were performed as described previously^8^. Samples were distributed across two membranes with three repetition hearts for inter-membrane calibration. All antibodies were obtained from Cell Signaling Technology (CST, Danvers, USA) with the exception of GAPDH, which was obtained from Santa Cruz Biotechnology (Sc, Dallas, USA). Protein expression was normalized to GAPDH.

| **Protein** | **Molecular weight** | **Catalogue number** |
| --- | --- | --- |
| ACC, anti-rabbit | 280 kDa | #3676 CST |
| Akt, anti-rabbit | 60 kDa | #9272 CST |
| AMPK, anti-mouse | 62 kDa | #2793S CST |
| AS-160, anti-rabbit | 160 kDa | #C699A7 CST |
| GAPDH, anti-mouse | 37 kDa | Sc-365062 |
| GSK3β, anti-rabbit | 47 kDa | #27C10 CST |
| pACC (Ser79), anti-rabbit | 280 kDa | #11818 CST |
| pAkt (Ser473), anti-mouse | 60 kDa | #4051S CST |
| pAMPK, anti-rabbit | 62 kDa | #4181P CST |
| pAS-160 (Thr642), anti-rabbit | 160 kDa | #42885 CST |
| pGSK3β (Ser9), anti-rabbit | 47 kDa | #9323 CST |

Table S2 Proteins used to assess key signaling molecules. Cell Signaling Technologies (CST), Santa Cruz Biotechnology (Sc), acetyl-CoA carboxylase (ACC), serine/threonine kinase (Akt), AMP-activated protein kinase α (AMPK-α), TBC1 domain family member 4 (AS-160), glyceraldehyde-3-phosphate dehydrogenase (GAPDH), glycogen synthase kinase 3β (GSK3β), phosphorylated acetyl-CoA carboxylase (pACC), phosphorylated serine/threonine kinase (pAkt), phosphorylated AMP-activated protein kinase α (pAMPK-α), phosphorylated TBC1 domain family member 4 (pAS-160), phosphorylated glycogen synthase kinase 3β (pGSK3β).

## **Gene expression**

mRNA expression was measured as described previously^9^. Ribosomal protein lateral stalk subunit P0 (RPLP0) and NADH ubiquinone oxidoreductase core subunit V1 (Ndufv1) were chosen as reference genes. The limit for replicate variability was set to 0.5 for the standard deviation. Relative quantities of the genes were calculated with the Quant Studio Design and analysis software (Thermo Fischer, Waltham, USA). Reference gene stability was verified as described in Hellemans *et al*. (2011)^10^. Sequences of the used primers are listed in Table S3:

| **Gene** | **Primer Sequence** |
| --- | --- |
| **CXCL-12** (NM_022177.3): | F: GCA TCG TCA TCC TGT CCT GT  R: ACG CTC TCG AAC TCA CAT CC |
| **CXCR-4** (NM_022205.3): | F: TCT GCA TCA GTG ACG GTA AGC  R: CTT CCT CAG GGG TCT ACT GGA |
| **IL-1β** (NM_031512.2): | F: AGGCTGACAGACCCCAAAAG  R: GGTCGTCATCATCCCACGAG |
| **IL-6** (NM_012589.2): | F: CCAGTTGCCTTCTTGGGACT  R: CTGGTCTGTTGTGGGTGGTA |
| **LDHA** (NM_017025.1): | F: ACCCTCTGGGGAATCCAGAA  R: TGGACCAACTGGACTAACCAC |
| **Ndufv1** (NM_001006972.1): | F: GCACAGCTGCGGTTATTGTTA  R: ATCCCCCTTCACAAATCGGG |
| **NFκb1** (NM_001276711.1): | F: TTCAACATGGCAGACGACGA  R: AGGTATGGGCCATCTGTTGAC |
| **TNFa** (NM_012675.3): | F: ATGGGCTCCCTCTCATCAGT  R: AAATGGCAAATCGGCTGACG |
| **PDK4** (NM_053551.1): | F: GAGCATCAAGAAAACCGCCC  R: CTTGGCGTAGAGACGGGAAA |
| **PGC-1α** (NM_031347.1): | F: GTG GAT GAA GAC GGA TTG CC  R: GGT GTG GTT TGC ATG GTT CT |
| **RPLP0** (NM_022402): | F: GCG ACC TGG AAG TCC AAC TA-3'  R: TTG TCT GCT CCC ACA ATG AA-3' |

Table S3 Sequence of forward (F) and reverse (R) primers for determination of gene expression: CXC-motive chemokine ligand 12 (CXCL-12), CXC-motive chemokine receptor type 4 (CXCR-4), interleukin 1 beta (IL-1β), interleukin 6 (IL-6), lactate dehydrogenase A (LDHA), NADH ubiquinone oxidoreductase core subunit V1 (Ndufv1), nuclear factor kappa light chain enhancer 1 (NFκb1), pyruvate dehydrogenase kinase 4 (PDK4), peroxisome proliferator activated receptor gamma coactivator-1α (PGC-1 α), ribosomal protein lateral stalk subunit P0 (RPLP0).

# **Supplementary results**

## **Macrophage polarization**

Macrophage phenotype was assessed through real-time PCR^11^, examining the upregulation of inducible nitric oxide synthase (INOS), indoleamine 2,3-dioxygenase (IDO), tumor necrosis factor alpha (TNF-α), C-C motif chemokine receptor 2 (CCR-2) and C-C motif chemokine ligand 11 (CCL-11).


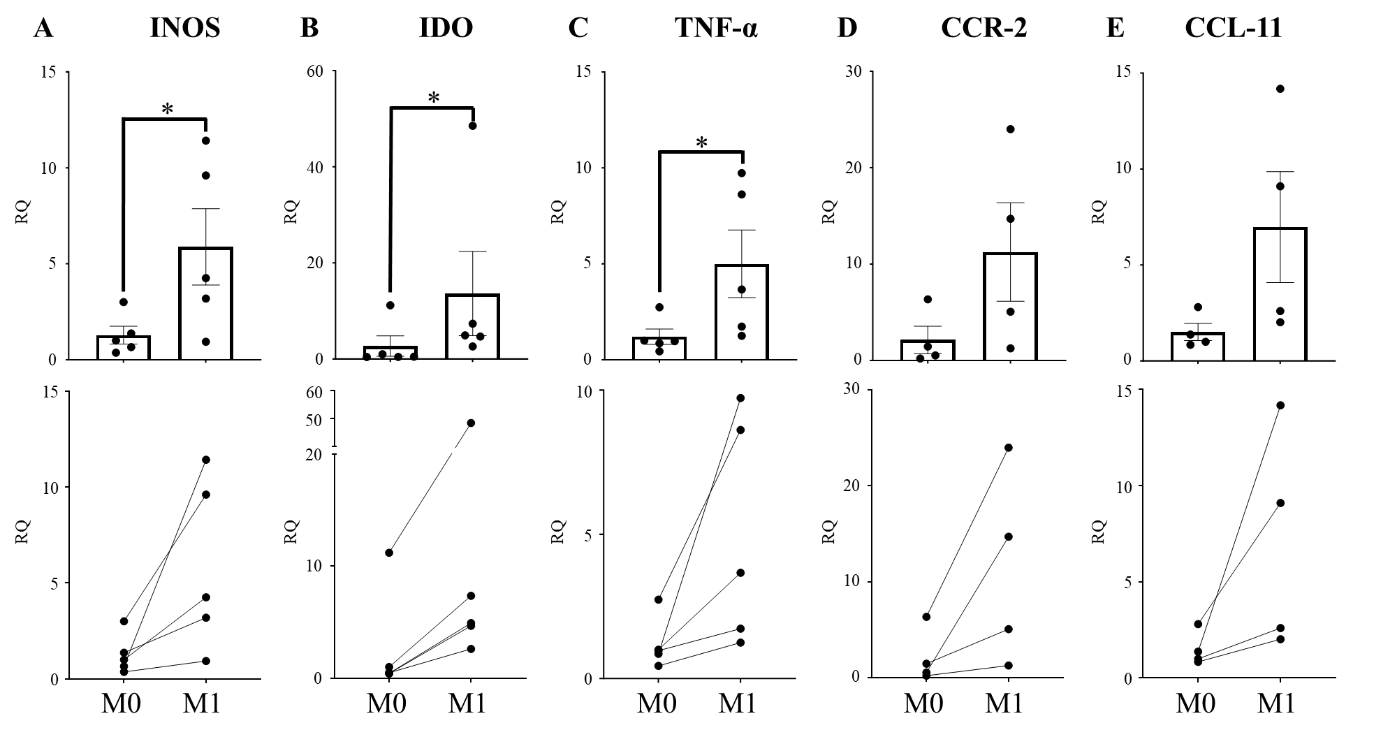


Figure S1 qPCR showing macrophage polarization (upper panel), increased expression after M1-polarization in different donors (lower panel) (A) Inducible nitric oxide synthase (INOS) (B) Indoleamine 2,3-dioxygenase (IDO) (C) Tumor necrosis factor alpha (TNF-α) (D) C-C motif chemokine receptor 2 (CCR-2) (E) C-C motif chemokine ligand 11 (CCL-11). Unpolarized macrophages (M0), pro-inflammatory macrophages (M1) *p <0.05, n = 4-5

## **EV characterisation**

NTA profile revealed EV size of approximately 100-300nm for both M0- and M1-EV. Western blots revealed expression of the EV specific markers; CD63, Alix, TSG101 and syntenin. Western blots were normalized by total protein concentration.


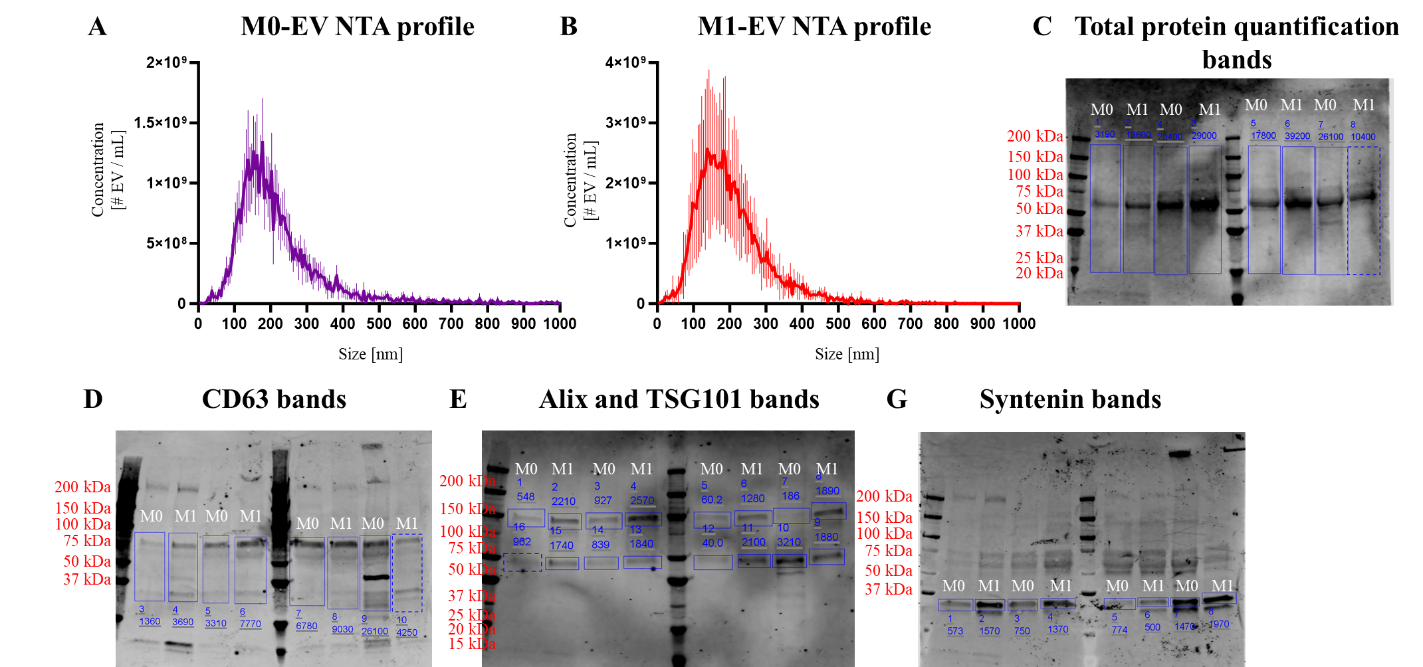


Figure S2 EV characterisation (A) NTA profiles for M0-EV (B) and M0-EV (C) total protein bands (D) CD63 bands (E) Alix bands at 95 kDa and TSG101 bands at 49 kDa, and (G) Syntenin bands. Nanoparticle tracking assay (NTA), extracellular vesicles (EV), unpolarized macrophages (M0), pro-inflammatory macrophages (M1).

## **Baseline characteristics**

Sample size and baseline characteristics for heart perfusions are shown in Table S4. No difference among experimental groups was observed, except for cardiac output and coronary flow. Cardiac output was significantly higher in No ISCH than in ISCH M0 and ISCH M1. Furthermore, the coronary flow was significantly higher in the No ISCH group compared to ISCH M1.

|  | **No ISCH** | **ISCH** | **ISCH M0** | **ISCH M1** |
| --- | --- | --- | --- | --- |
| **Number of hearts** | 6 | 6 | 6 | 7 |
| **Body weight [g]** | 407 [398-418] | 400 [401-403] | 394 [383-407] | 400 [391-410] |
| **Heart weight [g]** | 1.71 [1.69-1.72] | 1.68 [1.51-1.89] | 2.01 [1.59-2.07] | 1.76 [1.67-1.91] |
| **LV work [mmHg*beats/min]** | 34801 [32271-35836] | 32459 [31052-33609] | 32958 [30383-35218] | 30977 [29002-32304] |
| **Heart rate [beats/min]** | 256 [244-280] | 244 [236-257] | 263 [232-276] | 256 [246-268] |
| **Developed pressure [mmHg]** | 131 [131-136] | 133 [131-136] | 126 [122-134] | 123 [112-134] |
| **Maximal contraction rate [mmHg/sec]** | 4536 [4451-4668] | 4576 [4328-4856] | 4369 [3916-4793] | 4307 [3662-4920] |
| **Maximal relaxation rate [mmHg/sec]** | -3824 [-3651-  -4210] | -4001 [-3688-  -4346] | -3588 [-3278-  -3278] | -3647 [-3081-  -4055] |
| **Cardiac output [mL/min]** | 86 [85-88] | 79 [72-84] | 78* [68-86] | 76* [72-81] |
| **Coronary flow [mL/min]** | 38 [37-40] | 32 [29-34] | 33* [29-38] | 30 [27-33] |

Table S4 Baseline characteristics: LV work: left ventricular work (heart rate-developed pressure product). No ISCH: no ischemia without EV, ISCH: ischemia without EV, ISCH M0: ischemia with M0- derived EV and ISCH M1: ischemia with M1- derived EV. Data are expressed as median and interquartile range [IQR; 1^st^ - 3^rd^ quartile], *p <0.05 vs No ISCH.

## **Key signaling molecules in glucose metabolism**

Full western blot membranes are presented in Figure S3. Samples were distributed across two membranes with three duplicated hearts (repetitions) for inter-membrane calibration.


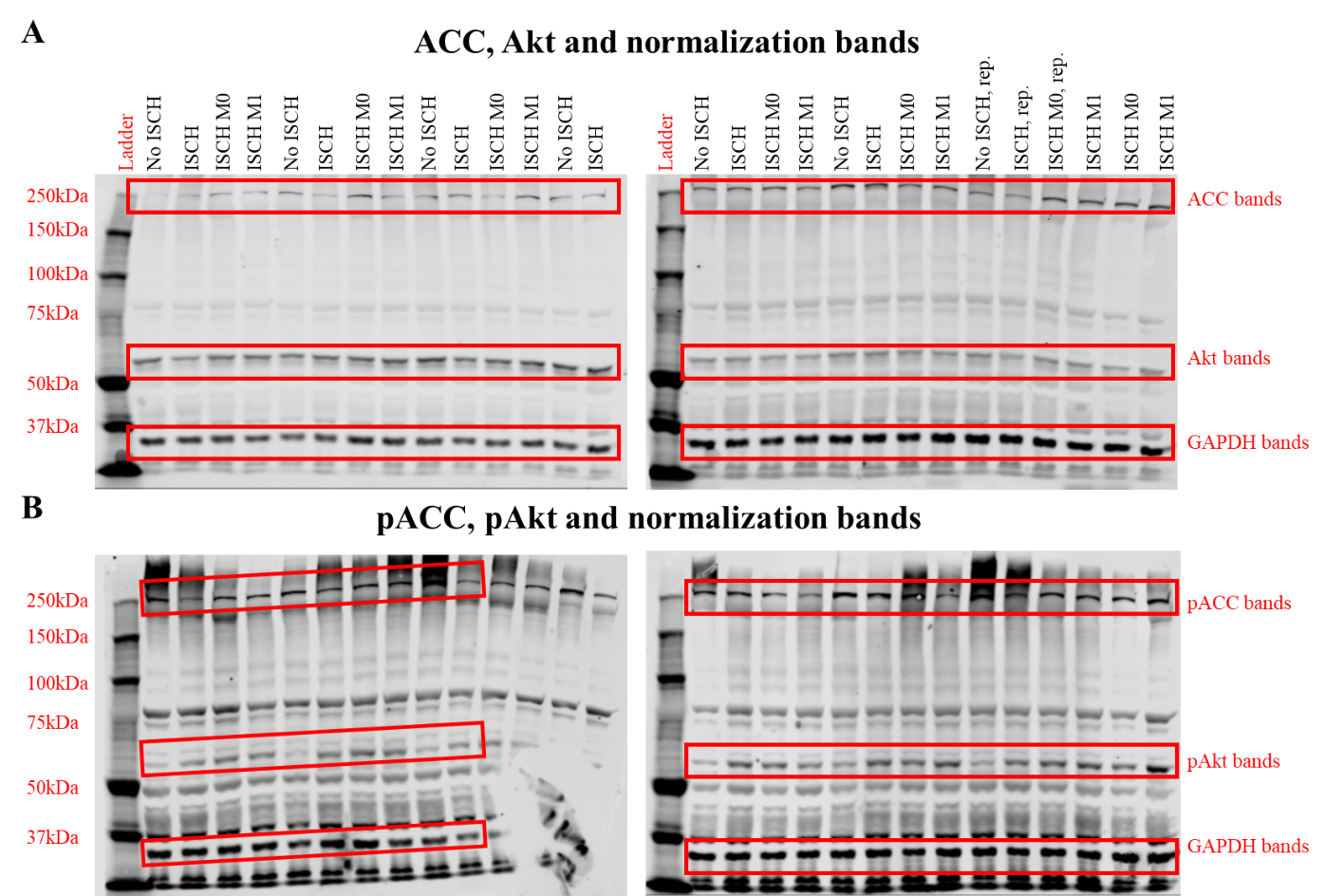


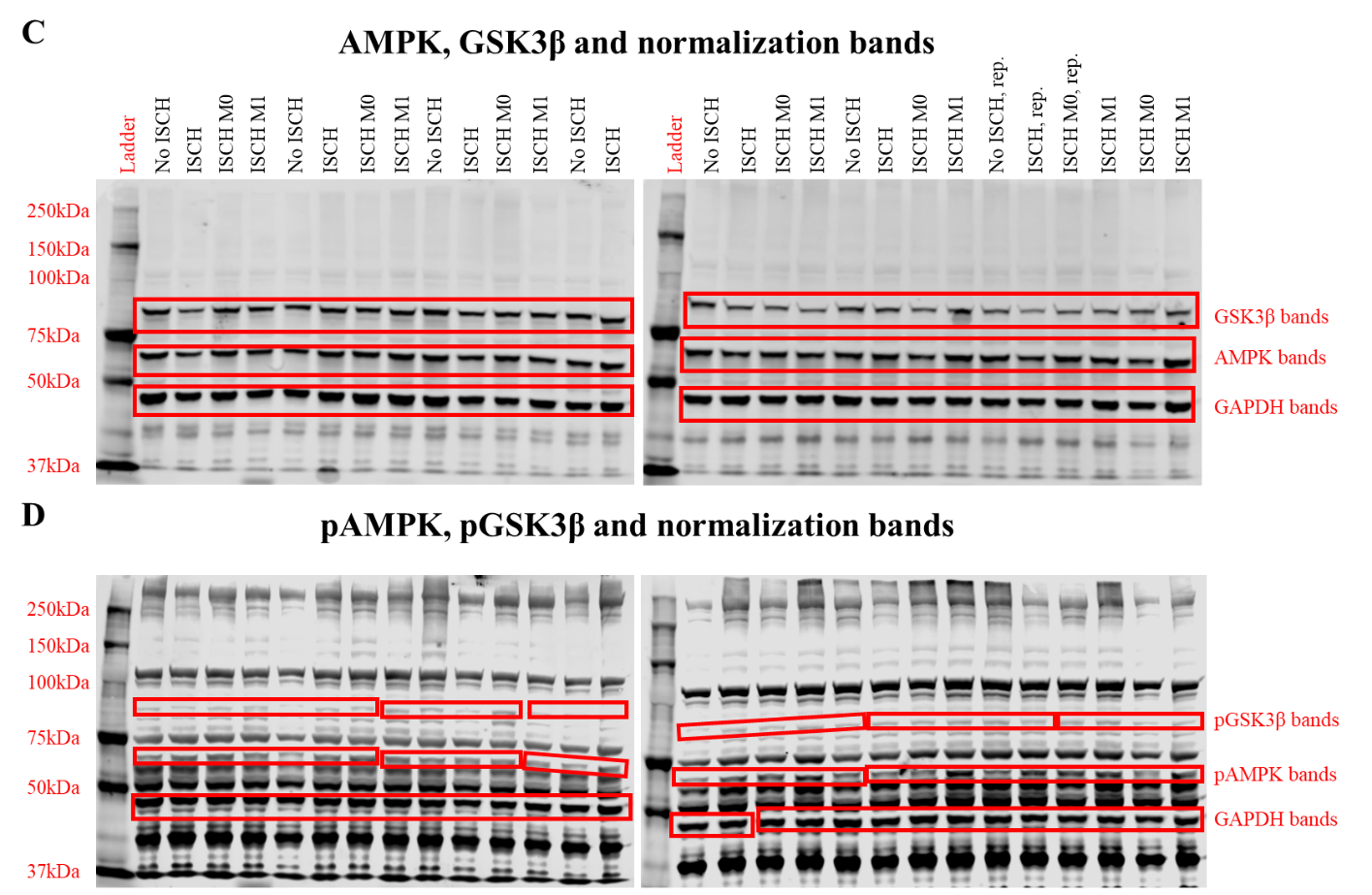


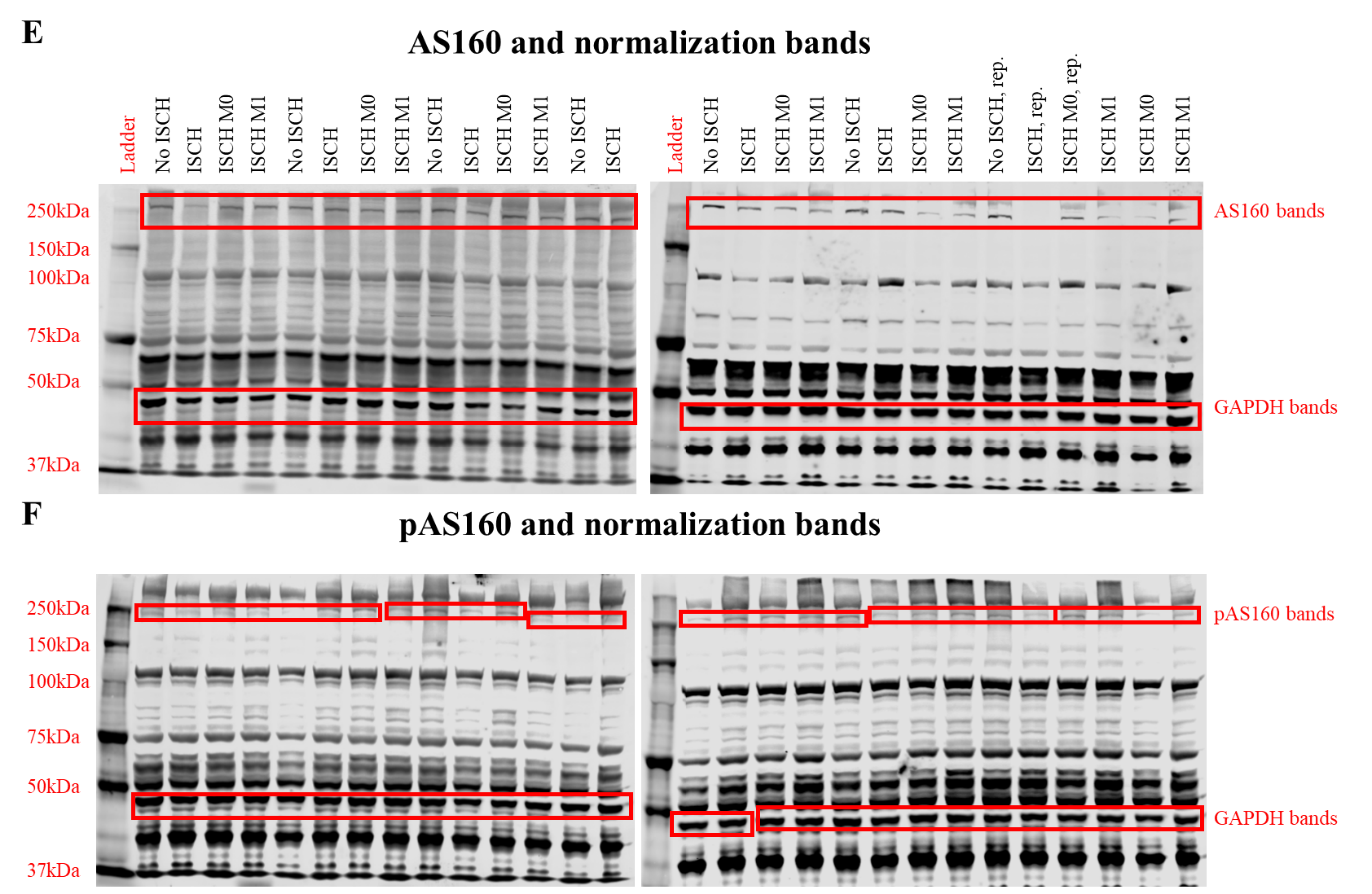


Figure S3 Uncut western blot membranes for total (Panels A, C and E) and phosphorylated (Panels B, D and F) proteins.

**Gene expression**

mRNA expression of interleukin-6 was similar among groups, while that of interleukin-1β and tumor necrosis factor alpha were lower in ischemic groups compared to No ISCH. No differences were observed between M0 and M1 for these genes. (Figure S2).

**
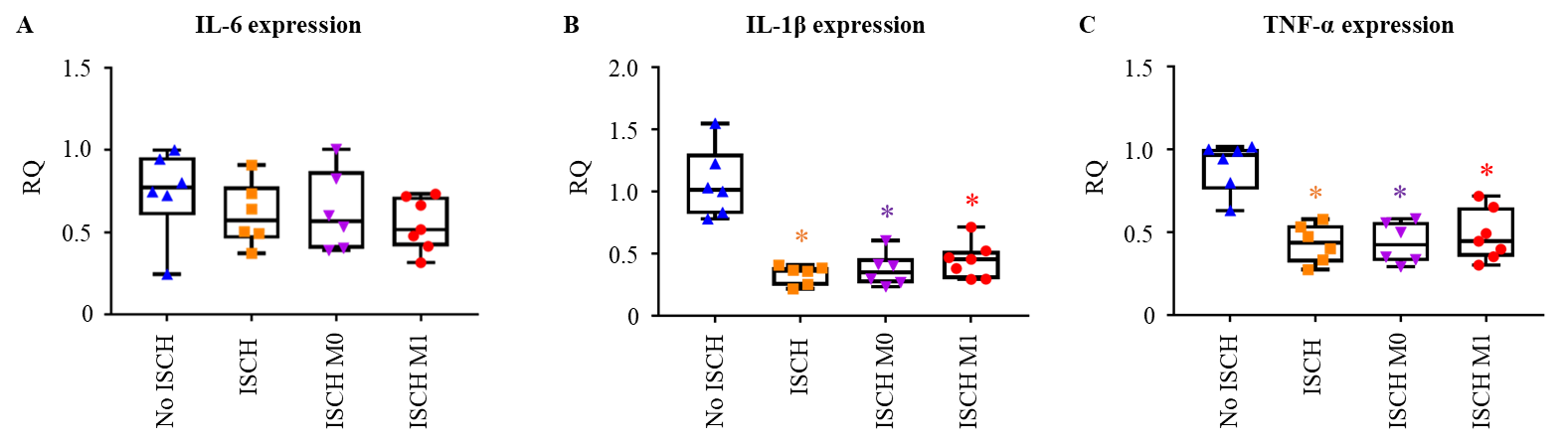
**

Figure S4 mRNA expression of cytokine genes at 60 minutes reperfusion (A) Interleukin 6 (IL-6) (B) Interleukin 1β (IL-1 β) (C) Tumor necrosis factor alpha (TNF-α). Gene expression is reported as relative quantity (RQ). No ISCH: no ischemia without EV, ISCH: ischemia without EV, ISCH M0: ischemia with M0- derived EV and ISCH M1: ischemia with M1- derived EV *p <0.05 vs No ISCH, #p <0.05 vs ISCH M1, n = 6-7 / group.

# **References**

1. Biemmi, V. *et al.* Inflammatory extracellular vesicles prompt heart dysfunction via TRL4-dependent NF-κB activation. *Theranostics* **10**, 2773–2790 (2020).

2. Popovic, M., Mazzega, E., Toffoletto, B. & De Marco, A. Isolation of anti-extra-cellular vesicle single-domain antibodies by direct panning on vesicle-enriched fractions. *Microb. Cell Factories* **17**, 6 (2018).

3. Akbar, N. *et al.* Endothelium-derived extracellular vesicles promote splenic monocyte mobilization in myocardial infarction. *JCI Insight* **2**, e93344 (2017).

4. Arnold, M., Méndez-Carmona N, Gulac P, Wyss RK, Rutishauser N, Segiser A, Carrel T, Longnus S., Rutishauser, N., Segiser, A., Carrel, T. & Longnus, S. Mechanical Postconditioning Promotes Glucose Metabolism and AMPK Activity in Parallel with Improved Post-Ischemic Recovery in an Isolated Rat Heart Model of Donation after Circulatory Death. Int J Mol Sci (2020). doi: 10.3390/ijms21030964. PMID: 32024002

5. Barr, R. L. & Lopaschuk, G. D. Direct measurement of energy metabolism in the isolated working rat heart. *J. Pharmacol. Toxicol. Methods* **38**, 11–17 (1997).

6. Niederberger, P. *et al.* High pre-ischemic fatty acid levels decrease cardiac recovery in an isolated rat heart model of donation after circulatory death. *Metabolism* **71**, 107–117 (2017).

7. Longnus, S. L., Wambolt, R. B., Parsons, H. L., Brownsey, R. W. & Allard, M. F. 5-Aminoimidazole-4-carboxamide 1-β- d -ribofuranoside (AICAR) stimulates myocardial glycogenolysis by allosteric mechanisms. *Am. J. Physiol.-Regul. Integr. Comp. Physiol.* **284**, R936–R944 (2003).

8. Méndez-Carmona, N. *et al.* Differential effects of ischemia/reperfusion on endothelial function and contractility in donation after circulatory death. *J. Heart Lung Transplant.* **38**, 767–777 (2019).

9. Sanz, M. N. *et al.* Cardioprotective reperfusion strategies differentially affect mitochondria: Studies in an isolated rat heart model of donation after circulatory death (DCD). *Am. J. Transplant.* **19**, 331–344 (2019).

10. Hellemans, J. & Vandesompele, J. *QPCR Data Analysis – Unlocking the Secret to Successful Results*. (Caister Academic Press, 2011). 978-1-904455-72-1

11. Biemmi, V. *et al.* Inflammatory extracellular vesicles prompt heart dysfunction via TRL4-dependent NF-κB activation. **10**, (2020).
